# Hepatocellular Carcinoma Differentiation: Research Progress in Mechanism and Treatment

**DOI:** 10.3389/fonc.2021.790358

**Published:** 2022-01-12

**Authors:** Jianning Song, Hongzhong Zhou, Dayong Gu, Yong Xu

**Affiliations:** ^1^Department of Clinical Laboratory, Institute of Translational Medicine, The First Affiliated Hospital of Shenzhen University, Shenzhen Second People’s Hospital, Shenzhen University, Shenzhen, China; ^2^Guangzhou Medical University, Shenzhen, China; ^3^Shenzhen Institute of Advanced Technology, Chinese Academy of Sciences, Shenzhen, China

**Keywords:** hepatocellular carcinoma, differentiation, liver-enriched transcription factors, tumor microenvironment, differentiation therapy

## Abstract

Hepatocellular carcinoma (HCC) is the most common primary malignant tumor of the liver. Although progress has been made in diagnosis and treatment, morbidity and mortality continue to rise. Chronic liver disease and liver cirrhosis are still the most important risk factors for liver cancer. Although there are many treatments, it can only be cured by orthotopic liver transplantation (OLT) or surgical resection. And the worse the degree of differentiation, the worse the prognosis of patients with liver cancer. Then it can be considered that restoring a better state of differentiation may improve the prognosis. The differentiation treatment of liver cancer is to reverse the dedifferentiation process of hepatocytes to liver cancer cells by means of drugs, improve the differentiation state of the tumor, and restore the normal liver characteristics, so as to improve the prognosis. Understanding the mechanism of dedifferentiation of liver cancer can provide ideas for drug design. Liver enrichment of transcription factors, imbalance of signal pathway and changes of tumor microenvironment can promote the occurrence and development of liver cancer, and restoring its normal level can inhibit the malignant behavior of tumor. At present, some drugs have been proved to be effective, but more clinical data are needed to support the effectiveness and reliability of drugs. The differentiation treatment of liver cancer is expected to become an important part of the treatment of liver cancer in the future.

## Introduction

Liver cancer is the sixth most common cancer disease globally, and the fourth leading cause of cancer death (1). Chronic liver disease and cirrhosis are the most important risk factors for liver cancer, among which viral hepatitis and excessive alcohol intake are the leading risk factors worldwide. Chronic diseases such as diabetes and obesity increase the risk of liver cancer. The study also found that the incidence in males is higher than that in females because of high testosterone levels (2).Hepatocellular carcinoma (HCC) accounts for more than 80% of primary liver cancer (3), it is one of the most common liver cancer with high morbidity and mortality. Molecular studies have identified mature hepatocytes as the origin cells of HCC. These cells dedifferentiate into hepatocyte precursor cells and then become HCC cells that express progenitor cell markers (4). The prognosis of most patients with liver cancer is poor, although monitoring patients with liver cirrhosis can be used to diagnose early liver tumors, but most of liver cancer are diagnosed in the late stage (5), this makes the treatment of liver cancer full of challenges. At present, only orthotopic liver transplantation (OLT) or surgical resection can cure (2), but the effect of therapy also depends on the size and location of the tumor and the state of the liver. Although chemotherapy and radiotherapy can prolong overall patients survival, the results are still not satisfactory because of tumor recurrence and drug resistance of cancer cells (6). Clinical treatment also found that poor prognosis of HCC patients with poor tumor differentiation (7), we can consider to restore the good differentiation state of the liver to achieve a better prognosis. We already know that dedifferentiation is a process of liver cancer development, so differentiation therapy whose main purpose is to induce and reverse tumor dedifferentiation may be a promising treatment strategy. Therefore, it is necessary to study the factors that change the expression level in the process of hepatocyte differentiation. Finding the changes and roles of these factors in the occurrence and development of liver cancer will help us to find more therapeutic targets. Tumor microenvironment (TME) is the cellular environment of tumor cells or tumor stem cells (8), the cells and molecules have many effects on the occurrence and development of tumor (9). In addition, a variety of inflammatory cytokines and regulatory pathways play a role in the dedifferentiation of hepatocellular carcinoma. Understanding these potential mechanisms can provide more insights into drug design. This article reviews the molecules that regulate the differentiation of hepatocellular carcinoma and some cells, cellular molecules, regulatory pathways and drugs that have been found to affect the progression of hepatocellular carcinoma.

## Liver Enriched Transcription Factors

**Figure 1:**
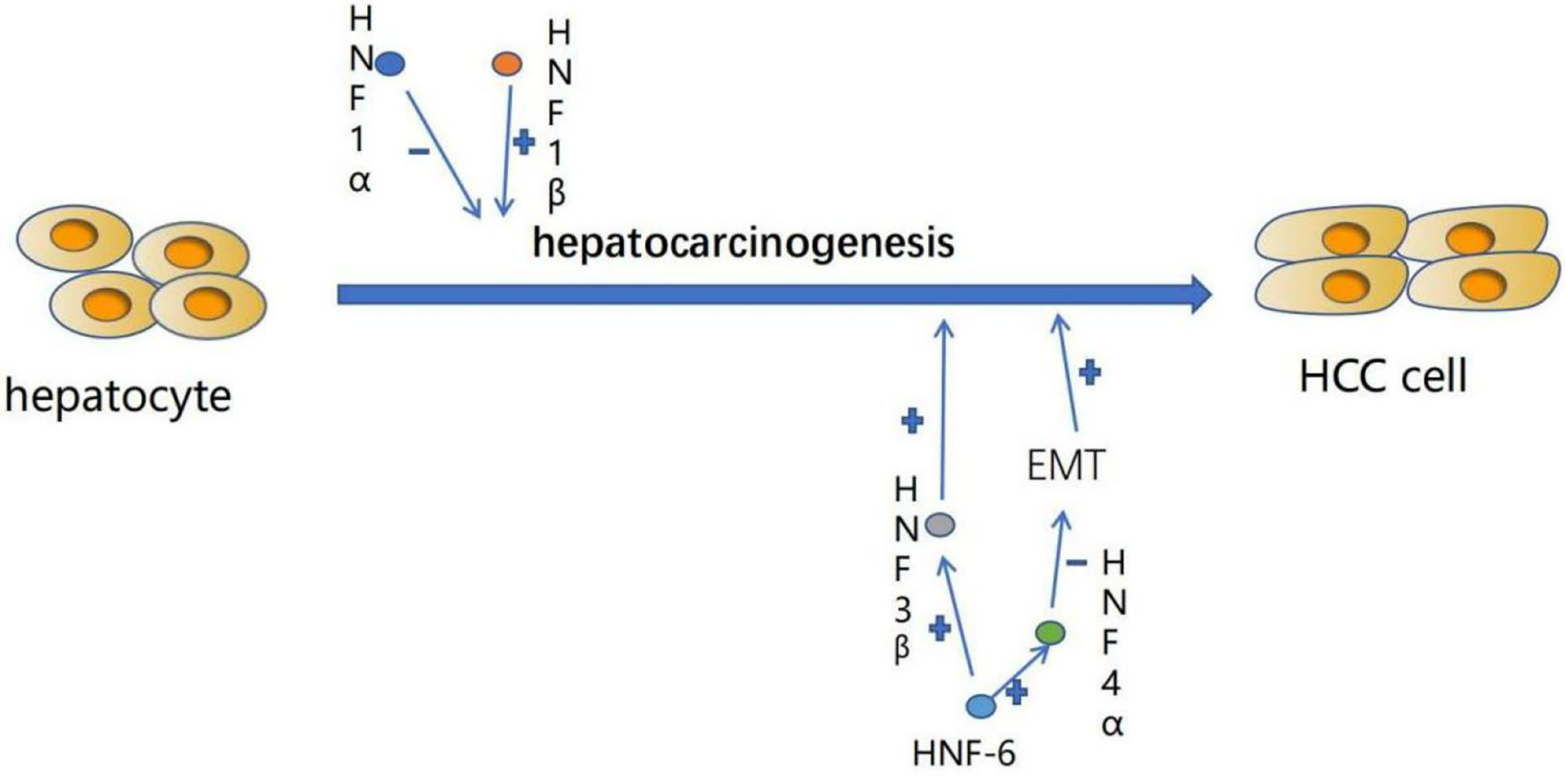
The expression level of liver-enriched transcription factors (LETFs) often changes during the occurrence of HCC, which can induce dedifferentiation of tumor cells and interfere with EMT procedures responsible for tumor progression. These characteristics can make LETFs a promising tool for HCC treatment. +:promotion. -: inhibition.

Hepatocyte differentiation is controlled by the combination of a variety of liver-enriched transcription factors (LETF), which are the key elements of liver-specific transcriptional regulatory genes. In the process of hepatocellular carcinogenesis, the expression level of these transcription factors often changes. Previous studies have shown that dedifferentiation of HCC is closely related to a large number of changes in transcription factor gene expression in the liver (**Figure 1**). This includes down-regulation of HNF4, HNF6, HNF1, HNF3 and C/EBP and up-regulation of COUP-TFI (**Table 1**) (18).

**Table 1:** The role of LETFs in the differentiation of liver cancer.

LETFs	Function	Mechanism	Ref.
HNF1α	Affect the growth and differentiation of liver cancer cells Differentiate liver cancer cells into hepatocyte-like cells	Inhibit the TGFb/Smads pathway Promote the endogenous expression of C/EBP and other hepatocyte nuclear factors	(10)
HNF1β	1.Promote the dedifferentiation of normal liver cells into cancer cells	Up-regulate the expression of Notch1 and Hes1 and activate the Notch1 pathway	(11)
HNF3γ	Promote differentiation of liver cancer cells Induce differentiation of hepatic stem cells	Increase the expression of hepatocyte-specific markers	(12)
HNF4	Induce redifferentiation of dedifferentiated liver cancer cells Participate in the differentiation of liver cancer	Activate HNF1α and liver genes (such as α-1-antitrypsin) CXCR7 inhibits HNF4 expression through ERK-dependent signaling	(13, 14)
HNF6	Promote the expression of HNF3β and HNF4 Inhibit the migration and invasion of liver cancer cells	Unknow	(15, 16)
C/EBP α	Dedifferentiation of hepatocytes into stem cell-like cells	Binding Ser193/Ser190 protein to dephosphorylate C/EBP α	(17)

The HNF1 family includes HNF1α and HNF1β. HNF1α and HNF1β proteins have similar homologous domains and POU domains, but the two transactivation domains of HNF1α in the C-terminal part of the proteins do not exist in HNF1β (19). Hepatocyte nuclear factor 1α is a key transcription factor in the HNF family, which interacts with DNA in the form of homologous or heterodimer with HNF1β. Both of them are involved in many important biological functions of hepatocytes, such as carbohydrate synthesis and storage, lipid metabolism, detoxification and serum protein synthesis. It also plays an important role in regulating liver development and hepatocyte differentiation (10). HNF1α is involved in hepatocyte differentiation and regulates most liver-specific genes at the transcriptional level (20). After hepatic parenchyma coagulation, HNF1α was preferentially expressed in the early stage of liver development, but decreased in adult liver (21). The expression level of HNF1α in well differentiated HCC tissues was higher than that in poorly differentiated HCC tissues (19), it is speculated that the high expression level of HNF1α in well differentiated hepatocellular carcinoma may be the result of early proliferation and differentiation of hepatocytes. The concentration of HNF1α protein in rat liver tumor decreased, and the binding activity of HNF1 protein decreased in the process of transformation from well differentiated human hepatocellular carcinoma to poorly differentiated human hepatocellular carcinoma (HCC), the binding activity of HNF1α protein decreased in the process of transformation from well differentiated human hepatocellular carcinoma to poorly differentiated human hepatocellular carcinoma (22). HNF1α promoter analysis confirmed that HNF4 was an important activator of HNF1α gene expression in addition to HNF1α self-regulation (15). Zengxin et al. confirmed that HCC was related to the decreased expression of HNF1α. They found that HNF1α could inhibit the proliferation of hepatocellular carcinoma cells, promote the expression of liver-specific genes in hepatocellular carcinoma cells, and abolish the tumorigenicity of hepatocellular carcinoma cells *in vivo (*10). It was also found that HNF1α could inhibit the activation of TGFb/Smads pathway in hepatocellular carcinoma cells and affect the growth and differentiation of hepatocellular carcinoma cells (10). It has been found that the simultaneous expression of HNF1α, HNF4α and FOXA3 can transform hepatoma cells into hepatocyte-like cells. Mechanically, the exogenous expression of HNF1α, HNF4α and FOXA3 in hepatocellular carcinoma cells promotes the endogenous expression of a variety of hepatocyte nuclear factors, including C/EBP, thus promoting the transformation of hepatocellular carcinoma cells into hepatocyte-like cells (23). On the contrary, the high expression of HNF1β enhances the tumor-forming ability of HCC cells *in vivo*, and promotes the dedifferentiation of hepatocellular carcinoma cells into liver cancer stem cells by activating Notch signal pathway, as well as the invasion of HCC cells and the occurrence of EMT (11). The expression of HNF1β can up-regulate the expression of Notch1 and Hes1 of genes related to Notch signaling pathway, activate Notch pathway, activate EMT and enhance the stemness expression of hepatocytes, and promote the dedifferentiation of normal hepatocytes to cancer cells, resulting in hepatocellular carcinoma (11). HNF1β can be used as an important predictor of poor prognosis of liver cancer (24).

The HNF3 family consists of HNF3α, HNF3β and HNF3γ.These proteins have high homology in the winged helix/forkhead DNA binding domain and two short similar regions at the C-terminal and N-terminal.HNF3 binds to other liver-enriched transcription factors and transactivates many liver-specific genes, such as albumin, transthyroxine, α 1-antitrypsin, and HNF1 α (15). HNF3 β, HNF3 α and HNF3 γ are activated successively during development (15). As a member of the HNF3 family, HNF3β exists mainly in the liver. Previously, it was found that HNF3β in HCC was up-regulated (25), but silencing the expression of HNF3β could inhibit the proliferation and invasion of HepG2 cells and promote apoptosis, while the overexpression of HNF3β had the opposite effect on HepG2 cells. At the same time, it also revealed that miR-141, as a cell proliferation inhibitor, plays an inhibitory role in HCC by inhibiting the expression of HNF3β (25). HNF3α plays an important role in maintaining hepatocyte differentiation (15). A recent study has shown that HNF3γ plays a role in maintaining hepatocyte differentiation. Further studies have found that delivery of HNF3γ to HCC cells can increase the expression of hepatocyte-specific biomarkers and enhance liver function, promote the differentiation of HCC cells, and overexpression of HNF3γ will also induce the differentiation of liver CSC. In addition, the authors also found that the differentiation of HCC cells mediated by HNF3γ led to the inhibition of HCC cell proliferation *in vitro* and the inhibition of HCC growth after xenotransplantation *in vivo (*12). These findings are of great significance for HNF3γ in the treatment of HCC differentiation.

HNF4 is expressed in the liver, kidney and intestine of adults (15). HNF4α is the main regulator of liver specific gene expression and has strong antitumor activity (26). HNF4 can transactivate endogenous HNF1α and liver genes (such as α 1-antitrypsin), and induce redifferentiation of dedifferentiated hepatoma cells by stably transfecting exogenous HNF4 (13). After HNF4 transduction, cells expressed previously silent HNF1α (15). HFN4α can inhibit the proliferation of hepatocellular carcinoma cells (26). The expression of HNF4α decreased during the occurrence of hepatocellular carcinom (27). At the same time, it was also found that the expression of HNF4α could inhibit hepatocyte EMT, and inhibit the formation of hepatoma stem/progenitor cells during carcinogenesis (27). The study also found that CXCR7 participates in HCC differentiation through ERK-dependent signal inhibition of HNF4α expression, and CXCR7-MAPK-HNF4α cascade is a general pathway of HCC differentiation. The authors believe that CXCR7-MAPK-HNF4α pathway can be used as a promising target for HCC differentiation therapy (14). HNF4γ2 is an isomer of HNF4α, which can restore dedifferentiated hepatoma cells to a more differentiated state by promoting the expression of hepatocyte markers (20).

HNF6 is expressed in tissues originating from endoderm cells of the liver, pancreas, foregut, nervous system, brain and spinal cord (15). It has been proved that the overexpression of HNF6 can stimulate the expression of HNF3β and HNF4 (15). HNF6 is a tumor suppressor. The expression level of HNF6 in hepatocellular carcinoma was negatively correlated with histological grade. The expression of HNF6 and differentiation-related markers in poorly differentiated hepatocellular carcinoma cells was lower than that in well differentiated hepatocellular carcinoma cells (16). HNF6 can up-regulate the expression of differentiation-related markers and inhibit the migration and invasion of hepatocellular carcinoma cells, while knockout HNF6 gene is the opposite (16).

C/EBP is abundant in the liver, especially in fully differentiated cells (15). C/EBP α and C/EBP β are involved in the proliferation and differentiation of hepatocytes (28). Tumor patients with high expression of C/EBP α or C/EBP β have a longer survival time (28). In human hepatocellular carcinoma, the expression level of C/EBP α is very low (22). The activity of C/EBPα is regulated by the phosphorylation of Ser193 (Ser190 in human protein), and the phosphorylation of C/EBP α at Ser193 site enhances its antitumor activity (17). The dephosphorylation of C/EBP α at Ser193/Ser190 can transform the tumor suppressor gene into a protein with carcinogenic activity, resulting in the transformation of hepatocytes into stem cell-like cells (17). In short, LETFs can induce tumor cell differentiation and interfere with the EMT program responsible for tumor progression, which makes LETFs a promising tool for HCC therapy.

## Tumor Microenvironment

**Figure 2:**
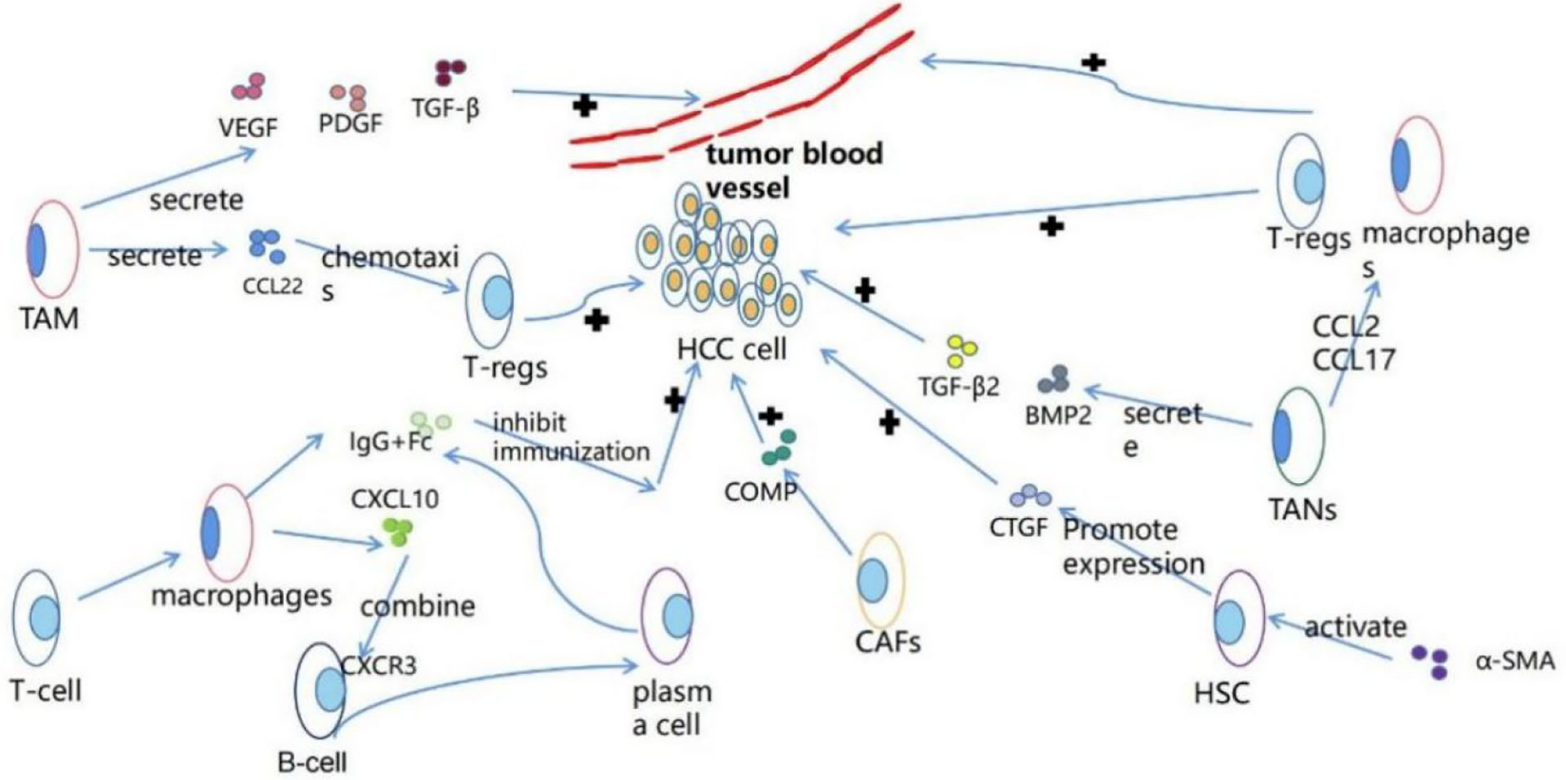
The functions of various types of cells in the tumor microenvironment.

Tumor microenvironment (TME) refers to the occurrence, growth and metastasis of tumor and the cellular environment of tumor cells or cancer stem cells. It is composed of malignant and non-malignant cells and a large number of soluble mediators (8). TME has a variety of effects on the occurrence, development and progression of tumors. It contains cells and molecules that can increase the stemness of tumor cells, promote angiogenesis, mediate migration, induce drug resistance and inhibit the immune system (**Figure 2**) (9). TME includes endothelial cells, fibroblasts and immune cells (9). Immune cells, such as granulocytes, lymphocytes and macrophages, participate in various immune responses and activities, such as inflammatory responses that promote tumor survival (9). Among them, macrophages play the most prominent role, which can promote tumor cells to escape to the circulatory system and inhibit anti-tumor immune mechanism and response (29). Studies have shown that macrophages participate in HCC cell-derived PKM2-mediated tumor microenvironment remodeling, which can promote the development of HCC. HCC-derived exosomal PKM2 not only induces metabolic reprogramming of monocytes, but also induces nuclear STAT3 phosphorylation and upregulates the expression of differentiation-related transcription factors, leading to differentiation from monocytes to macrophages and tumor microenvironment remodeling (30).

Tumor-associated macrophage (TAM) is an important part of tumor microenvironment (31). Hepatic macrophages are composed of different ontogenetic populations, namely Kupffer cells and monocyte-derived macrophages (Mo-Mfs). At present, macrophages have been designated as classical activated M1 state triggered by interferon-γ and alternative activated M2 state induced by lipopolysaccharide or IL-4. TAM is usually defined as M1-like (leading to anti-tumor response and cytotoxicity) or M2-like (tumor promotes and suppresses effective adaptive immunity) cells, that is, M2 TAM has tumor-promoting effect, while M1 TAM has anti-tumor effect (32). TAM can produce a variety of chemokines, such as CCL22, can attract regulatory T cells to the cancer site, thus blocking the activation of cytotoxic T cells (33), to achieve immunosuppression. TAM can also produce angiogenic factors such as vascular endothelial growth factor (VEGF), platelet-derived growth factor and transforming growth factor-β to promote tumor angiogenesis (31). Hepatocytes are epithelial cells with highly specialized morphology. Epithelial-mesenchymal transformation (EMT) and HCC dedifferentiation describe the same thing to some extent, but their emphasis is different (20). Recent studies have shown that tumor microenvironment is closely related to epithelial-mesenchymal transformation of cancer cells, and the interaction between them can promote epithelial-mesenchymal transformation in cancer cells (34), tumor-related macrophages, especially M2-type macrophages, can produce EMT-promoting cytokines, such as IL-6 (35), it can be considered that TAM may promote HCC dedifferentiation. TNF- α released by M2 macrophages can promote the epithelial-mesenchymal transformation and cancer stemness of HCC, indirectly promote the dedifferentiation of hepatocellular carcinoma cells and promote the progress of HCC (36).

Neutrophils are typical immune cells, which can promote cell transformation, tumor progression and anti-tumor immunity in tumor microenvironment (37). Tumor-associated neutrophil (TANs) plays an important role in promoting tumor progression in tumor microenvironment (38). In HCC cells, TANs can secrete BMP2 and TGF- β 2, increase the expression of miR-301b-3p, and increase the stem cell characteristics of HCC cells (38), which may promote the dedifferentiation of HCC. In the mouse model, TANs recruited macrophages and Treg cells through the expression of CCL2 and CCL17 in HCC, promoted their infiltration in tumor, and promoted HCC neovascularization and HCC progression (39).

Fibroblasts are spindle-shaped slender cells. More and more evidence shows that fibroblast subsets can regulate the progression of cancer. These cells are called cancer-associated fibroblasts (CAF) or tumor-associated fibroblasts (TAF) (40). CAF may come from hepatic stellate cell (HSC), epithelial mesenchymal transformed (EMT) parenchyma cells, bone marrow (BM)-derived cells, mesothelial cells and portal vein fibroblasts (PF) (41). CAFs can maintain and enhance the dryness of HCC cells (42), it can secrete cartilage oligomeric matrix protein (COMP) to promote the proliferation, invasion and EMT, of HCC cells as well as tumorigenesis and growth *in vivo (*43). Studies have shown that IL-6 secreted by CAF may enhance the stemness of HCC cells through Notch signaling pathway (44). Matrix niche can regulate the differentiation and proliferation of stem cells by providing a unique microenvironment. CAF, as a part of the surrounding matrix in TME, can increase the stem cell-like characteristics of cancer cells by secreting growth factors such as HGF, thus regulating the differentiation of cancer stem cells (45). The differentiation of liver cancer cells remains to be studied.

Hepatic stellate cell (HSC) is an important part of HCC tumor microenvironment. Activated HSC can be transformed into myofibroblast-like cells to promote liver injury or chronic inflammatory fibrosis, leading to liver cirrhosis and HCC (46). The expression of α-SMA (a marker of HSC activation) can promote the expression of connective tissue growth factor (CTGF), and CTGF-mediated tumor-matrix interaction between hepatocellular carcinoma cells and hepatic stellate cells can promote the progression of HCC (47). Recent studies have shown that overexpression of miR-1246 or knockdown of ROR α can activate and promote epithelial-mesenchymal transformation of (EMT) through Wnt/β-catenin pathway to promote HCC progression, while miR-1246 is triggered by HSC, and ROR α is the target gene of miR-1246 (48). Recently, it has been reported that hepatic stellate cells can undergo mesenchymal-epithelial transformation (MET), restore to epithelial cells, and eventually differentiate into hepatocytes or bile duct cells (**Table 2**) (49).

**Table 2:** The role of cells in cancer differentiation.

Cell	Function	Mechanism	Ref.
M2 macroph-ages	Promote epithelial-mesenchymal transition of liver cancer Promote the stemness of liver cancer	Release tumor necrosis factor-α Produce cytokines that promote EMT	(35, 36)
TANS	Enhance the stem cell characteristics of liver cancer cells	Secrete bone morphogenetic protein 2 and transforming growth factor-β2, increase the expression of miR-301b-3p	(38)
CAF	Increase the stem cell-like properties of cancer cells and regulate the differentiation of cancer stem cells	Secrete growth factors such as HGF	(45)
HSC	Differentiation into hepatocytes or bile duct cells	Mesenchymal-Epithelial Transition (MET)	(49)

B cells can be found at the invasive edge of the tumor, and are more common in draining lymph nodes and lymphoid structures near TME (50). Human HCC tissue contains B cells. Activated CD4+T cells from HCC stimulate macrophages to produce CXCL10;CXCL10 that binds to CXC chemokine receptor 3 on B cells, making them plasma cells that produce IgG. IgG activates Fc receptors in macrophages to produce cytokines that reduce anti-tumor immune response, thus promoting the progress of HCC (51).

Tumor microenvironment is the environment for the growth of cancer cells. Many components of tumor microenvironment play a role in the differentiation of liver cancer, and also provide direction for the differentiation and treatment of HCC. The importance of TME in the design of new cancer treatment programs is now obvious.

## Inflammatory Cytokines

**Figure 3:**
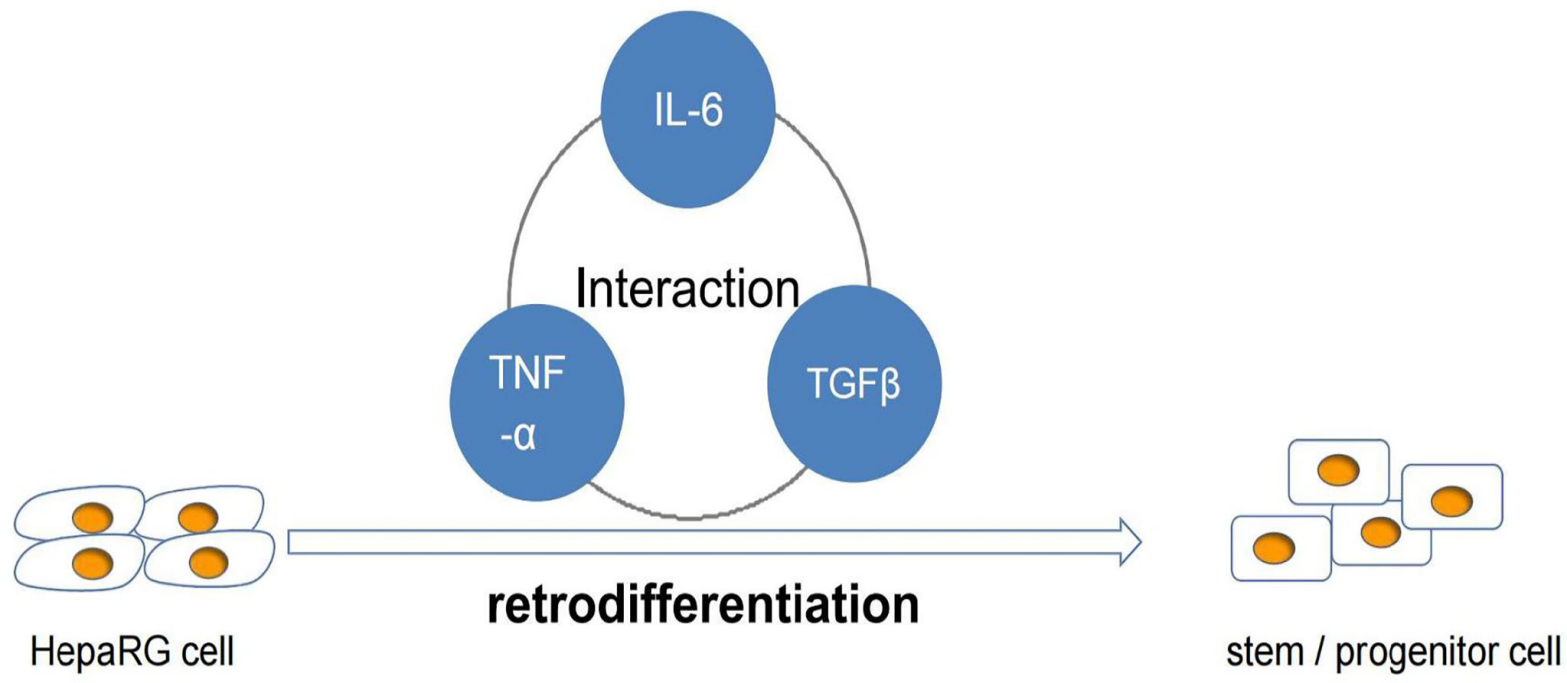
Role of IL-6 in differentiation of hepatocellular carcinoma.

Inflammation can mediate the development of HCC, which involves a variety of cytokines. IL-6 can activate STAT3 signal pathway to drive hepatocyte replication and promote the occurrence of hepatocellular carcinoma (52). Studies have shown that the production of IL-6 is necessary for the growth and malignant transformation of HCC progenitor cell (HcPCs). Hepatocellular carcinoma (HCC) progenitor cells (HcPC) are precancerous cells. The increased expression of LIN28 leads to the secretion of IL-6, which makes HcPC differentiate into hepatocellular carcinoma cells (53). STAT3 can also be activated by IL-17, and the promoting effect of IL-17 on HCC is through the activation of IL-6/STAT3 pathway (54). IL-6 can promote the retrodifferentiation of tumor-derived HepaRG hepatocyte-like cells (HepaRG-tdHep) into proliferative stem/progenitor cells through the interaction between TNF–α and TGF-β. Tumor necrosis factor-α activates IL-6 through NF- κ B pathway and phosphorylates STAT3 at the same time. On the other hand, IL6 plays many roles by activating STAT3. There is crosstalk between transforming growth factor-β and interleukin-6 pathway, which contributes to the EMT of liver cancer cells and promotes the dedifferentiation of liver cancer cells so as to promote the progression of liver cancer (55). It is also discussed in the literature that TGF β can induce EMT in malignant hepatocytes by stimulating the proliferation of CAFs (**Figure 3**) (56).

Studies have shown that interferon-γ (IFN-γ) can significantly increase the apoptosis of HCC cells, significantly up-regulate the expression of Bax and lytic caspase-3 in HCC cells, and down-regulate the expression of Bcl-2. Interleukin-17 can inhibit the apoptosis of HCC cells by inhibiting the induction of IFN-γ, thus promoting the progress of HCC (57). NK cells trigger CSCs/undifferentiated tumor differentiation mainly through secretion and membrane-bound IFN-γ and TNF-α, and IFN-γ plays a greater role (58). It was also found that IL-10 inhibits NK cell-mediated tumor differentiation by inhibiting IFN-γ secretion during monocyte-mediated NK areactive induction (59). Interleukin-33 (IL-33) is an effective pro-inflammatory cytokine inducer, which can regulate innate immunity and adaptive immunity (60). Current studies have found that up-regulation of IL-33 in HCC leads to accelerated growth of HCC, and IL-33 may induce chemokines (such as Cxcl1) to enhance the recruitment of S100A9-secreting bone marrow cells to promote tumor progression (60). IL-34 is a cytokine produced by a variety of cells, which is initially involved in controlling the differentiation, proliferation and survival of bone marrow cells (61). In tumor microenvironment, IL-34 mediates the interaction between tumor cells and TAM (61). especially in hepatocellular carcinoma, tumor cell-derived IL-34 can stimulate TAM to produce transforming growth factor β 1 (TGF-β 1), inhibit the expression of miR-28-5p on HCC cells (61), promote the differentiation and proliferation of TAM, and indirectly promote the differentiation and progression of HCC (61).

A series of studies on the above molecules can greatly promote our understanding of the mechanism of cancer development and differentiation, and provide new insights into the methods and targets of differentiation treatment.

## Treatment Status

At present, when patients have symptoms of HCC, the diagnosis of liver cancer is often in the late stage of the disease, which is very difficult for treatment and prognosis. And at this later stage, there are few effective treatments that can improve survival (2). Orthotopic liver transplantation (OLT) is the best treatment choice for patients with decompensated liver cirrhosis, and HCC is the only solid tumor that can be treated with transplantation (2). However, OLT is only applicable to patients who meet the Milan criteria or the University of San Francisco criteria. Non-operative methods include transarterial chemoembolization (TACE), which is the standard treatment for hepatocellular carcinoma in the middle stage (62), and it is also the most commonly used initial treatment for local hepatocellular carcinoma (63). For unresectable liver cancer, the 1-year and 2-year survival rates of TACE treatment were 82% and 63%, respectively (64). Other methods include percutaneous local ablation, microwave ablation, cryoablation, radiotherapy and systemic chemotherapy (2). The therapeutic effect and selection of the above methods depend on the size, location, extrahepatic spread and potential liver function of the tumor, because the diagnosis of liver cancer is often in the late stage, and in order to improve the therapeutic effect and postoperative survival rate, new treatments need to be studied to achieve a better prognosis.

The concept of cancer differentiation therapy probably originated in the 1970s, when the first *in vitro* model system was developed to culture cells and induce them to differentiate with specific drugs. The purpose of inducing differentiation is to limit the growth potential of cancer cells. Many different biological mechanisms are thought to explain the positive therapeutic effects of inducing cancer cell differentiation *in vivo*. It includes inducing irreversible loss of proliferative potential, inhibiting the reactivity of growth factors, inhibiting tumor angiogenesis, inducing and inhibiting the expression of oncogenes, enhancing the effect of chemotherapy and radiotherapy drugs and inducing antigens to be recognized by cytotoxic immune cells (65). At present, there are three ways to treat cancer differentiation in theory: (1) targeted differentiation of cancer; in targeted differentiation of cancer, the differentiation pathway is activated without correcting the potential carcinogenic mechanism that leads to the block of initial differentiation. (2) reverse differentiation of cancer; in the process of tumor reverse differentiation, correcting the potential carcinogenic mechanism leads to the recovery of endogenous differentiation pathway. (3) tumor metastasis and differentiation: in the differentiation of cancer metastasis, cancer cells are redirected to the early stage of differentiation to obtain alternative differentiation pathways (66). Recently, targeting CSC is becoming an important therapeutic strategy. Differentiation therapy may induce CSCs to become terminally differentiated cancer cells. These cells can then be treated with alkylated antineoplastic agents (such as cyclophosphamide and busulfan) or a combination of both therapies for better results (67).

**Figure 4:**
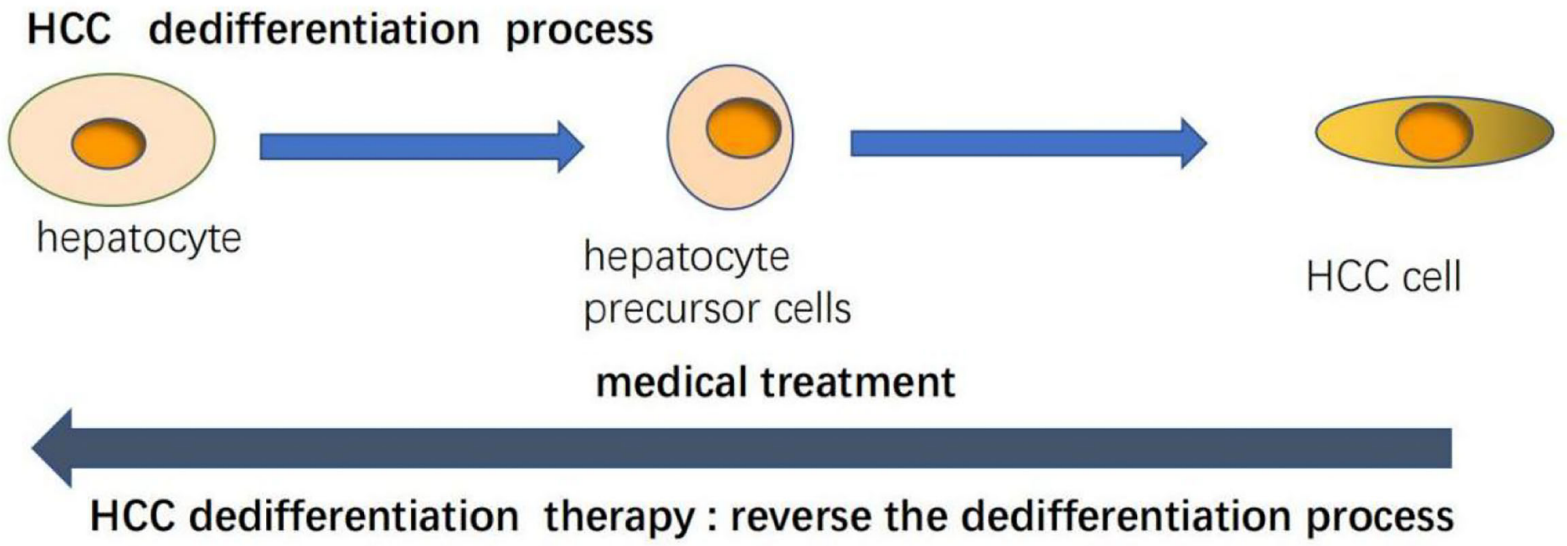
HCC differentiation therapy aims to reverse HCC dedifferentiation process.

As we already know from the above, there are a variety of molecules that can regulate and induce the differentiation of liver cancer, restore the normal level of these molecules and inhibit tumor behavior. The differentiation therapy of liver cancer is based on such a basis, that is, the main goal is to reverse the dedifferentiation process from liver cells to liver cancer cells, improve the differentiation state of the tumor and restore the characteristics of the liver (**Figure 4**). There are now a variety of drugs that can achieve this goal (**Table 3**). All-trans retinoic acid (ATRA) is a group of structural and functional analogues of vitamin A, which can regulate the growth, differentiation and development of epithelial cells (68). Studies have found that ATRA is a good differentiation inducer, which can induce the differentiation of a variety of tumor cells. ARTA has been used clinically in the treatment of patients with acute promyelocytic leukemia (69). At the same time, it was also found that the hepatoma cells treated with ATRA showed better differentiation characteristics. ATRA can induce the down-regulation of CD147 and promote the differentiation of HCC cells, and also show the up-regulation of differentiation marker HNF4 (70). Studies have shown that ARTA treatment decreased the protein level of β-catenin in CD133+ liver cancer stem cells, and increased the protein phosphorylation of β-catenin. At the same time, it was also found that the knockdown of β-catenin mRNA decreased the protein expression of stem cell markers in CD133+ liver cancer stem cells and damaged their dryness (81). Sorafenib is an oral multienzyme inhibitor that inhibits tumor angiogenesis and cell growth by inhibiting the activities of serine/threonine kinases c-Raf (Raf-1) and B-Raf; mitogen-activated protein kinases mek and erk; vascular endothelial growth factor receptor (Vegfr)-1, 2 and 3; platelet-derived growth factor receptor (Pdgfr)-α and β as well as c-kit, Flt-3 and Ret (82). ATRA can enhance the efficacy of sorafinib (20). Studies have shown that cisplatin may be sensitive to the treatment of hepatocellular carcinoma in children (83). ATRA can effectively induce the differentiation of tumor initiation cells and enhance the cytotoxicity of cisplatin, thus enhancing the chemotherapeutic effect of cisplatin on HCC (71). As2O3, like ATRA, has been used in the clinical treatment of acute promyelocytic leukemia (72). As2O3 can restrict the growth of hepatoma cells, down-regulate the anti-apoptotic protein Bcl-xL, and up-regulate the expression of Notch, leading to apoptosis (73). Studies have shown that As2O3 down-regulates the expression of CD133 and some dry genes to induce differentiation of HCC cancer stem cells. The authors further found that As2O3 can induce differentiation of HCC cancer stem cells, inhibit recurrence and prolong survival after hepatectomy by targeting GLI1 expression (74). As2O3 can also inhibit the growth of hepatocellular carcinoma by up-regulating Mir-1294 expression (75). In addition, As2O3 inhibits MCM7 transcription by targeting serum reaction factor (SRF)/microchromosome maintenance protein 7 (MCM7) complex, thus inhibiting the function and metastasis of liver cancer stem cells (84). As2O3 can also induce HCC cell death through TNF-related apoptosis-inducing ligand (TRAIL) signaling pathway in combination with sorafenib (85). Recent studies have also found that As2O3 can reduce the expression of NF- kappa B by inducing DNA demethylation to activate microRNA-148a, thus inhibiting CSC-like phenotype, which may provide a new therapeutic mechanism for HCC (76). Tumor inhibin M (OSM) is a cytokine associated with interleukin 6 (IL-6) produced by CD45+ hematopoietic cells. It is a multifunctional cell regulator that acts on a variety of cells and plays a potential role in the regulation of gene activation, cell survival, proliferation and differentiation (77). Moreover, OSM is a tumor-related cytokine highly expressed in patients with liver cirrhosis and HCC, which can regulate the accumulation of macrophages, and TNF-α derived from macrophages mediates the activation of hepatic progenitor cells. OSM overexpression can accelerate the occurrence of liver cancer, and play an important role in the occurrence of liver cancer by regulating liver inflammatory environment (78). OSM can be used as a potential target for prevention and treatment of HCC. Previous studies have found that OSM can induce hepatocyte differentiation of EpCAM+HCC. OSM treatment increased the chemical sensitivity of EpCAM+HCC cells, especially when OSM combined with 5-FU significantly inhibited the proliferation of tumor cells (79). Other studies have found that OSM can induce the differentiation of HCC cell line SMMC-7721 cells and reduce their cell viability, which also shows that the differentiation therapy of OSM can provide new opportunities for the treatment and intervention of HCC (77). It has been found that DNMT1 silencing significantly reduces DNA methylation in cells and can promote the differentiation of HCC cells (80). 5-AZA is a specific inhibitor of DNMT1 (86), and its epigenetic repair can improve the cytotoxic effect of sorafenib on HCC cells (80). Moreover, the experimental mice treated with 5-AZA also showed their ability to inhibit HCC cells and restore liver differentiation (80). The mammalian target of rapamycin (mTOR) signaling pathway has received considerable attention because of its key role in cell growth control. It includes two different complexes, mTORC1 and mTORC2 (87). Moreover, the mTOR pathway is abnormally activated in human HCC (88). Everolimus and Ku0063794 are inhibitors of mTOR. The combination of ivermus and Ku0063794 can more effectively inhibit the proliferation, migration and invasion of HCC cells. At the same time, it can also inhibit the EMT effect of HCC cells by inhibiting the regulatory factor SIRT1 protein that promotes HCC, so as to achieve the anticancer effect (87). These drugs have a variety of functions, the most important one is that they can promote differentiation and inhibit the malignant behavior of liver cancer cells, and when combined with other drugs, they can improve drug sensitivity and achieve better therapeutic effects. and to make up for the limitation that the current systemic therapy can not completely destroy liver cancer cells (20). Of course, because of the complex differentiation process and the different reaction mechanism of each cancer cell, a specific differentiation agent can not act on all cells in cancer, which needs to be further studied in clinical practice.

**Table 3:** Drugs for differentiation and treatment of HCC.

Drugs	Function	Mechanism	Reference
ATRA	Promote cell differentiation Damage cell stemness Enhance the efficacy of sorafinib	Down-regulation of CD147, up-regulation of HNF4. Reduce the protein level of β-catenin and increase its phosphorylation	(68, 69)
Solafini	Inhibition of tumor angiogenesis Inhibit cell growth	Inhibition of serine/threonine kinase activity Inhibition of protein kinase mek and erk activity Inhibition of vascular endothelial growth factor receptor activity Inhibition of platelet-derived growth factor receptor activity	(70)
As2O3	Inhibit the growth of hepatocellular carcinoma cells Induce differentiation of cancer stem cells Inhibit CSCs function	Down-regulation of anti-apoptotic protein Bcl-xL, Up-regulation of Notch signal expression Up-regulation of Mir-1294 expression. Inhibition of MCM7 transcription Down-regulation of the GLI1 expression	(71–75)
Oncostatin M	Inducedifferentiation of hepatocellular carcinoma cells Inhibit the proliferation of tumor cells	Unkown	(76, 77)
5- AZA	Promote HCC cell differentiation Improvethe cytotoxicity of sorafenib	1.Silence DNMT1, reduce DNA methylation	(78, 79)
Everolimus +Ku0063794	Inhibition of proliferation, migration and invasion of HCC cells Inhibition of EMT effect of HCC cells	1.Inhibitory regulatory factor SIRT1 protein expression	(80)

## Conclusions and Perspectives

Hepatocellular carcinoma (HCC) is still the leading cause of cancer death worldwide because of its high morbidity and mortality and advanced liver dysfunction. Although there are many ways to treat the disease to improve the survival rate of patients, there are still many limitations for patients, whether liver transplantation or chemotherapy, especially in HCC patients who have advanced to advanced stage. Systemic treatment is a more optimal treatment for advanced patients, which can improve the survival rate of patients. Sorafenib, as a systemic drug, is currently a first-line treatment for advanced patients. Even so, the high recurrence rate of HCC patients still leads to a poor prognosis, so it is urgent to solve the problem of poor prognosis to improve the survival rate of patients. After the successful application of differentiation therapy for acute promyelocytic leukemia, the differentiation therapy for HCC has also been proposed. Differentiation therapy aims to use drugs to induce cancer cells to differentiate into benign cells or even normal cells. It can be used as a new treatment for HCC to reduce the recurrence rate and improve the prognosis. In recent years, the study of molecules and signal pathways regulating the dedifferentiation of hepatocellular carcinoma is helpful to the development of new drugs (20). Although most of them are still in the experimental stage and no phase III clinical trials have been conducted, exciting results have been achieved and it is expected to further identify the target population of these drugs. At the same time, more data is needed to support the safety and effectiveness of this treatment. Differentiation therapy is expected to become an important part of HCC treatment in the future. And the treatment methods for HCC are also evolving with the research. At present, some studies have found that different immunotherapy or immunotherapy combined with other methods to treat HCC has a higher survival rate and a better prognosis (89). We believe that after continuous clinical experimental research, there is expected to be a more perfect treatment for the treatment of HCC.

## Author Contributions

JS wrote the first draft of the manuscript. HZ wrote section of the manuscript and provided suggestions for revision. YX and DG contributed to conception of the manuscript. All authors contributed to manuscript revision, read, and approved the submitted version.

## Funding

This work was supported by China Postdoctoral Science Foundation (Grant No. 2020M682910), and Shenzhen Science and Technology Foundation (Grant No. JCYJ20210324103407018), Guangdong Science and Technology Foundation (Grant No. 2021A1515110931).

## Conflict of Interest

The authors declare that the research was conducted in the absence of any commercial or financial relationships that could be construed as a potential conflict of interest.

## Publisher’s Note

All claims expressed in this article are solely those of the authors and do not necessarily represent those of their affiliated organizations, or those of the publisher, the editors and the reviewers. Any product that may be evaluated in this article, or claim that may be made by its manufacturer, is not guaranteed or endorsed by the publisher.
